# Perspectives on econometric modelling to inform policy: a UK qualitative case study of minimum unit pricing of alcohol

**DOI:** 10.1093/eurpub/ckt206

**Published:** 2013-12-22

**Authors:** Srinivasa V. Katikireddi, Lyndal Bond, Shona Hilton

**Affiliations:** 1 MRC/CSO Social and Public Health Sciences Unit, University of Glasgow, 4 Lilybank Gardens, Glasgow, G12 8RZ, UK; 2 Public Health and Health Policy, NHS Lothian, Waverley Gate, 2-4 Waterloo Place, Edinburgh, EH1 3EG, UK; 3 Centre of Excellence in Intervention and Prevention Science, 15-31 Pelham Street, Carlton South 3053, Australia

## Abstract

**Background**: Novel policy interventions may lack evaluation-based evidence. Considerations to introduce minimum unit pricing (MUP) of alcohol in the UK were informed by econometric modelling (the ‘Sheffield model’). We aim to investigate policy stakeholders’ views of the utility of modelling studies for public health policy. **Methods**: In-depth qualitative interviews with 36 individuals involved in MUP policy debates (purposively sampled to include civil servants, politicians, academics, advocates and industry-related actors) were conducted and thematically analysed. **Results**: Interviewees felt familiar with modelling studies and often displayed detailed understandings of the Sheffield model. Despite this, many were uneasy about the extent to which the Sheffield model could be relied on for informing policymaking and preferred traditional evaluations. A tension was identified between this preference for *post hoc* evaluations and a desire for evidence derived from local data, with modelling seen to offer high external validity. MUP critics expressed concern that the Sheffield model did not adequately capture the ‘real life’ world of the alcohol market, which was conceptualized as a complex and, to some extent, inherently unpredictable system. Communication of modelling results was considered intrinsically difficult but presenting an appropriate picture of the uncertainties inherent in modelling was viewed as desirable. There was general enthusiasm for increased use of econometric modelling to inform future policymaking but an appreciation that such evidence should only form one input into the process. **Conclusion**: Modelling studies are valued by policymakers as they provide contextually relevant evidence for novel policies, but tensions exist with views of traditional evaluation-based evidence.

## Introduction

Obtaining *a priori* evidence for population-based interventions can be difficult, as there is often a lack of *post hoc* evaluations in many areas of public health policy.^1^^,^^2^ In response, there is increasing interest in the use of mathematical modelling to inform decisions about population-based public health interventions.^3^^,^^4^ Previous research on policymakers’ views of improving the use of evidence in public health policy has similarly suggested the need for more studies that predict the effects of interventions.^5^ However, there is currently a lack of evidence about how policymakers understand and make use of modelling studies. Alcohol minimum unit pricing (MUP) policy presents the opportunity to investigate stakeholders’ views of modelling evidence in a real-life public health policy context.

MUP for alcohol is a relatively novel policy intervention that ensures alcohol products are not sold below a minimum price, determined by their alcohol content. The policy aims to reduce alcohol-related harms by preventing sales of cheap alcohol, thereby preventing supermarkets engaging in loss-leading to increase footfall into their stores, and seeks to target drinkers at greatest risk of harm. However, critics have argued that the measure is regressive and may result in unintended consequences, such as illicit alcohol sales.

The Scottish and the UK governments have been debating the introduction of MUP as a response to escalating health and social harms in their respective jurisdictions.^6^ The Scottish government has passed legislation to introduce the measure, with implementation delayed as a result of legal challenges.^7^^,^^8^ In contrast, the UK government has abandoned plans to adopt MUP.^9^ Both governments have been informed in their deliberations by econometric modelling conducted by the University of Sheffield (hereafter referred to as the ‘Sheffield model’) and more recently, applied internationally.^10^

The Sheffield model was originally commissioned by the UK government’s Department of Health to model the impact in health, crime and economic terms of a number of different policy options including a ban on below-cost sales, a ban on off-sales promotions, increases in alcohol duty and MUP (with a range of different levels for the price per unit considered).^11–13^ Other relevant research that became available during the policy debates derived from evaluations of reference pricing (which sets minimum prices that vary by beverage type in some Canadian provinces).^14^^,^^15^

The Sheffield model operates through two linked components—an econometric model and an epidemiological model.^11^ The econometric component (referred to as the ‘price-to-consumption’ model) relates policy interventions to price changes and hence consumption changes by calculating ‘elasticities’, which measure how purchasing of a product changes in response to a price change.^11^ Importantly, the model operates in a sophisticated manner by allowing for considerable heterogeneity in responses to policy interventions.^16^ This includes allowing for responses to differ by population subgroups (e.g. by age, gender and drinker type, i.e. moderate, hazardous or harmful consumption and ‘binge’ drinker status) and different alcoholic products (e.g. beer, wine, spirits and alcopops). The population subgroups were chosen in conjunction with policymakers to allow the modelling of effects on specific groups of interest, such as young ‘binge’ drinkers who have been considered a policy priority within the UK government. The epidemiological component (also referred to as the ‘consumption-to-harm’ model) takes these estimated consumption changes and relates them to outcomes of interest (e.g. hospital admissions, crimes, mortality) in a deterministic manner based on population attributable fractions.^11^ By using routinely available national data, the Sheffield team has been able to predict the harms prevented as a result of implementing different policy options. For example, the model predicts that MUP set at 50 pence per unit level would reduce consumption by 5.7% and 8600 hospital admissions per year.^13^

Results of the Sheffield model have been highly influential in the policy debate. In particular, the model has helped those advocating for policy change by demonstrating that the introduction of a MUP is likely to be a more targeted intervention than other policy options (such as increasing alcohol duty).^17–19^ In other words, those who drink hazardously and harmfully are most likely to consume cheaper alcohol and are therefore affected by the policy to a greater extent than those who drink moderately. While there has been broad support for MUP among non-industry groups, alcohol-related industries have been more split.^20^

Although the use of modelling is not unique in policy terms,^21^ the use of such a complicated model to explicitly predict the impact of different policy options is unusual for public health policy.^3^ There have been calls for research to better understand how those involved in policy debates respond to this form of modelling evidence.^16^ In this article, we aim to describe how those involved in Scottish and the UK policy debates perceived modelling evidence, including the Sheffield model, and describe their views on the potential future role of similar modelling within public health policy.

## Methods

Thirty-six one-to-one semi-structured interviews were conducted with individuals who had been directly involved in the UK- and/or Scotland-based policy debates on MUP. Potential participants were identified from an initial stakeholder analysis (drawing on publicly available UK and Scottish Parliamentary documents), supplemented by snowball sampling. Participants were purposively chosen to create a diverse sample in terms of two key factors: level of supportiveness for MUP and the sector worked in. Additionally, diversity was sought within each sector (e.g. seeking the inclusion of politicians from different political parties; civil servants from various departments both in Scotland- and Westminster-based Governments; industry actors working in producer, off-license and trade organizations).

Interviews were guided by an interview schedule that included questions on how the Sheffield model was perceived, the influence of the Sheffield model on the policy debate and the future role of modelling studies in public health policy. Interviews typically lasted between 45 min and 1 h. Interviews were conducted face-to-face (n = 23) or over the telephone (n = 13) by the lead author (S.V.K.). Contemporaneous fieldwork notes were made.

The limited number of potential participants for this study increases the risk of interviewee identification and could therefore threaten recruitment. To improve the potential for recruitment and the quality of data obtained, a tiered process for obtaining informed consent was pursued.^22^ In addition to seeking consent for being interviewed (required for participation), separate permission was sought (and obtained for most participants) for the following: audio recording, the use of quotations in publications and presentations and identification of the broad sector the participant was drawn from (i.e. politician, civil servant, researcher, advocate and industry). Following the interview, and in conjunction with interviewees, small sections of transcripts were either marked not for quotation or minimally reworded to prevent disclosure of participants’ identities (e.g. removal of names or references to organizations).

Following transcription, interview data were read repeatedly and analysis proceeded in keeping with the principles of grounded theory.^23^ Inductive thematic coding was conducted by S.V.K., with initial descriptive codes created and subsequently recoded to characterize emergent themes. The principle of the constant-comparative method was used to help identify explanations for patterns within the data while also paying appropriate attention to contradictory data.

### Ethical Approval

The study was reviewed and obtained ethical approval from the University of Glasgow’s College of Medicine and Veterinary Science research ethics committee.

## Results

Eight academics, seven advocates, ten civil servants, six industry representatives and five politicians were interviewed. The results present a number of important themes identified from the data in turn.

### Understanding of the Sheffield model

In general, respondents were familiar with the notion of modelling to inform decision-making and frequently drew on their previous encounters with what they saw as similar modelling exercises to the Sheffield model. A diverse range of comparisons was used by interviewees including the introduction of the minimum wage in the UK, infectious disease modelling in relation to outbreaks (specifically pandemics), regulatory impact assessments (that require potential impacts of policy to be assessed in advance of implementation) and modelling the obesity burden. Despite this awareness of other examples of modelling, it did seem to a number of respondents that the Sheffield model represented something qualitatively different from their previous encounters—in the words of one advocate, ‘it’s slightly different but […] it doesn’t feel too dissimilar’.

Respondents, and especially those based in Scotland where the policy debate was more advanced, typically showed a high level of knowledge about the results and methodology of the Sheffield model. For example,

Politician: ‘And of the 18– 24-year olds—which, if we go back to the public perception of the night economy—they are the least affected by this measure. […] you look the figures up and it’s 1.6%. So 23 units a year less at 45 pence. That’s half a pint a week. Come on, you know, what the hell is that doing?’

It is worth noting that this quotation comes from a verbal discussion in which the respondent had no written documents with him/her and so had (accurately) committed detailed figures from the Sheffield model to memory.^24^

### The Sheffield model as knowledge

Despite interviewee familiarity with the idea of modelling, there was considerable debate about the extent to which the Sheffield model constituted legitimate knowledge that could inform decision-making and whether it should be considered ‘evidence’ (see table 1a).

**Table 1:** Tensions in econometric modelling as a form of public health evidence

Theme	Illustrative quotation
1a) Econometric studies as ‘not research’	Industry: I think it [the Sheffield model] consistently is referred to as evidence, consistently is referred to as research, and it’s closer to research than evidence. There was undoubtedly a large research base behind it but it is effectively a model. So you know, people refer to the ‘Sheffield research, Scharr’s Research’. No, the ‘Sheffield evidence’ or ‘Scharr’s evidence’ when you know, the two terms should not be used in the same sentence; it’s modelling.
1b) Econometric studies as ‘just modelling’	*Interviewer: You mentioned the Sheffield kind of modelling work. What do you think of the use of modelling work to kind of inform policy debate?*
	Academic: (Laughs). Well I like the little platitude of ‘do you believe the weather forecast? That’s modelling’. You take data, you use it, you try to make your best guess based on the relationships and trends you can see. You try to make the best predictions from that. I’m in sympathy with people who say ‘it’s just modelling’. And therefore I think the only answer can come from running the experiment and the Scottish government has been very courageous to run the experiment.
1c) Econometric studies as different from other public health evidence	Civil Servant: I mean, if it hadn't been, you know, if we hadn't had the Scharr reports then, you know, we'd have got nowhere. And of course we had lots of debates about the extent to which it was evidence because it was modelling …
1d) Econometric studies in tension with epidemiological training	Academic: When politicians and journalists ask you for your opinions, ‘well maybe they really want to hear my opinions’ and I did get a bit carried away and felt that I had been unfaithful to my scientific training because I suddenly felt that I really did believe that minimum unit price was going to be a good thing. Whereas to be honest, we don’t know. We don’t know. We’ve got models. Sheffield modelling etc, all the taxation stuff but we don’t know. And we don’t know what’s gonna happen to the very heavy, heavily dependent drinkers. We actually don’t know and there may be some pluses and minuses.
1e) Econometric studies as having high external validity	Academic: And mostly researchers […] just say, ‘well, this policy was introduced and it didn’t work or it did work’, and then the policy-maker looks at that and says, ‘well, that was then, and that wouldn’t necessarily apply here and now’. ‘Well, you know, that [evaluation] was done over there in Australia or Canada …’ they never believe it would work. So something that’s done locally, using local data, UK data, and at the request of Government, that’s what needed to happen. That’s why it was effective.

Many interviewees expressed the view that modelling, while helpful, was imperfect and subordinate to other forms of academic knowledge (table 1b). There, therefore, appears to be an important distinction made between what might be considered more conventional forms of evidence (such as trials and evaluations) from the type of econometric modelling exemplified by the Sheffield study (table 1c). The somewhat ambiguous status of modelling studies led to active discussion among some actors more familiar with more frequently used forms of public health evidence, such as epidemiological studies and evaluations (table 1d). On some occasions, this led to a tension for public health professionals between maintaining a commitment to being ‘evidence-based’ (as underpinned by studies of effectiveness of public health interventions) and their responsibility as public health advocates.

However, while most interviewees expressed a preference for more conventional forms of evidence, the benefits of evaluation studies over *a priori* modelling were not always considered so clear-cut. Perhaps the most obvious indication of this is the relatively little emphasis placed by interviewees on the Canadian experiences (which included evaluation studies that demonstrated a decrease in consumption and harms following the introduction of the related policy intervention of reference pricing). In contrast to the detailed awareness respondents had of the Sheffield studies, an interviewee in favour of MUP referred to the Canadian studies as ‘something relevant’ while in contrast rating the Sheffield models as hugely important in making the case for MUP.

Another tension was evident between what was considered the need for gold-standard evidence in the form of evaluation studies and the applicability of research from elsewhere. Econometric modelling was, therefore, valued as providing highly applicable evidence that related closely to the policymaking context (see table 1e).

### Predicting intervention effects in a complex system

Debates about the extent to which the Sheffield model could help understand a system as complex as the alcohol market were common. Before considering specific issues raised by respondents, it is worth noting that many interviewees (including some sceptical of MUP) felt that the Sheffield team had made a good attempt at engaging with the different dimensions requiring consideration by policymakers.

That said, concerns were expressed about the extent the Sheffield model related to current ‘real life’ (table 2a). In other words, the adequacy of the baseline scenario within the model was questioned for not accurately capturing the current realities of alcohol sales or the changes in the market over time. A second area of concern revolved around the extent that the Sheffield model considered important changes in the alcohol market that may occur in response to policy changes (table 2b). Interviewees critical of MUP while suggesting these issues should have been taken into account, also tended to express dissatisfaction with a perceived lack of transparency within the model—an issue likely to be made more difficult if the model incorporated greater complexity.

**Table 2:** The adequacy of the Sheffield model in capturing complexity

Theme	Illustrative quotation
2a) Adequacy in capturing baseline complexity	Industry: […] they didn’t model what would happen if that drove consumption to, from England or to online. And yet we look at online and every single week is the, is a record week for online sales. Every week for about the last six months we’ve sold more this week than we did last week through the internet on everything including alcohol.[…] We will deal with much larger variances than, than we see [in the model]. And therefore it becomes, it’s quite risky for us to put all of our faith into that. So, what role would we use for it? Well, I mean we have looked at it, we’ve looked at it in terms of how might that change consumption but we take it with a pretty big dose of salt. We wouldn’t take any business decisions on that. We don’t think it’s robust in the real world. Because it doesn’t, it just doesn’t take into account those other factors.
2b) Adequacy in modelling complex effects of MUP	Academic: So I think that, you know, one particular critique of the Sheffield approach is that they don’t really allow for second round effects of minimum pricing. So how does it feed through on the industry side. Now of course that’s probably an order of magnitude more difficult to model than what happens on the consumer side. But I think perhaps trying to sort of come up with some scenarios where you would say well in the case where there’s a knock-on effect on other alcohol prices go up, this is what happens; in the case where there’s a knock on effect on other alcohol prices come down, this is what happens. There are economic models that you can estimate that would allow you to try and predict what you think the industry response would be under some assumptions about how the industry behaves, and I haven’t seen any of that in the debate. And you know, perhaps it would be a nice thing to try and do. It’s again complicated and it’s limited by the data that we do and don’t have at our disposal but I think that could have been a feature of the debate.

### Communicating uncertainty

The importance of communication was repeatedly emphasized. Many interviewees suggested that the uncertainties inherent in the modelling exercise were frequently not adequately communicated (table 3a). However, those actually responsible for communicating findings from the Sheffield model were clearly aware of the risks in presenting the Sheffield model in too certain terms but also suggested communication of risk in general, and econometric modelling in particular, was difficult (table 3b).

**Table 3:** Communicating uncertainty—necessary but difficult?

Theme	Illustrative quotation
3a) Uncertainty has not been communicated adequately in the policy debate	Academic: I do sometimes think that perhaps a little too much certainty is place on the results of the modelling. So when you look at a lot of the discourse from supporters of minimum pricing in Scotland where they talk about the policy leading to X number of saved lives in year one or fewer admissions or whatever, you know, it’s worth kind of bearing in mind that there’s a huge amount of uncertainty around those estimates. I don’t expect ministers to say you know 40 fewer deaths plus or minus 35 but it would be nice to have some acknowledgement that this is based on model estimates without it coming over as this will definitely happen because I think it leaves you open to possible criticism if it doesn’t happen.
3b) Communicating ‘risk’ is generally difficult, modelling even more so	Civil Servant: So, yeah, trying to explain modelling and, you know, elasticities and all of that, I mean, I find it difficult to get my head around that, so, you know, not surprising that that's quite a difficult thing to explain to the public, media, you know, committee, especially when people don't necessarily want to believe it either, you know? […] but I guess it's like all of these things that, you know, we're not very good, we're not very literate with uncertainties and, you know, like we always say about risk, you know, people find it really hard to get their head round …

In addition, there was an awareness that some individuals (especially politicians) need to be able to communicate findings from the Sheffield model in a politically charged environment to potentially hostile audiences (such as parliament or mass media) and this could be challenging. Some interviewees noted that in this case, the fact that the Sheffield model had resulted in a clear message (that MUP was a targeted intervention) had helped communication efforts, and future modelling studies may not result in as simple messages.

### The future for modelling public health policy options

In general, there was considerable support for increased use of modelling to inform public health decision-making among all interviewees (table 4a). There was an appreciation among those involved in public health on an ongoing basis that because modelling required specific expertise, this may require collaborative work with econometricians or statisticians. However, a number of interviewees expressed caution at the idea of actively advocating for increased modelling. On the one hand, several interviewees highlighted the need for better evaluation of policy interventions and were keen modelling was not seen as a substitute for such work (table 4b). On the other hand, some interviewees highlighted the risk that a lack of modelling (or evidence in general) might impede action when needed (table 4c)—a point echoing the concerns of being narrowly ‘evidence-based’ discussed earlier.

**Table 4:** Views on the future use of econometric modelling to inform public health policy

Theme	Illustrative quotation
4a) More econometric modelling to serve as a laboratory for population-based interventions	Academic: Well, yes, [there is a need for more modelling] because a lot of things that we might talk about influencing public health – particularly at population level – are things where they’re not necessarily amenable to randomized controlled trials and, therefore, modelling is a stage that you would go to before you would go to the intervention. So if you think about it in terms of other kinds of interventions, you know, you don’t develop a new drug and take it straight onto the market. You go through stages of testing – is it safe and is it effective, etc.? So modelling is kind of a public health laboratory, in some respects, so you can test what the predicted effects of a policy will be, look at these consequences of being right or wrong, look at the confidence around the effects before you make the case for implementing it in real life.
4b) Econometric modelling is helpful, but evaluation is the priority	*Interviewer: Do you think there’s the potential for a greater role for modelling studies elsewhere in public health?*
	Academic: Yes, I’d sort of say this with slight nervousness. I think one of the biggest problems … I mean yes is my short answer to that. But I think one of the most, … the most important issue is that there are so many policy changes that go on that are just not properly evaluated and there’s no doubt that everyone is much happier with a real life experience, well evaluated, than a model; so I think you need both. […] the biggest gap is that there’s so many policy changes go on that are just not evaluated
4c) More econometric modelling is helpful, but should not be a barrier to taking action when necessary	Advocate: […] I’m a very, very big advocate for evidence-informed policy, I’m also of the view that sometimes if the evidence is not there, or it’s grey, then you invoke the precautionary principle. So, you know, modelling research has its place, and it’s a useful tool, I don’t think it needs to be the key tool, and equally I don’t think that we should get too caught up and not be prepared to do anything unless there’s compelling evidence, which is not always the case.

Despite the general enthusiasm for the increased use of modelling, the importance of allowing for value judgements was emphasized by politicians. In addition, modelling studies were weighed up against other forms of knowledge, including an individual’s own experiences and observations, which they felt were more grounded in real life. For example, another politician critical of MUP said the following:

[…] to be perfectly honest, you know, with all these studies, you know, and I hope you’ll take this in the spirit in which it’s intended in, but you know, I’ve never been a big one who’s – in terms of being blinded by some study that’s been carried out in an ivory tower somewhere. I mean, I try to think of what I call sort of logic and human nature and my observation of human nature over a period of time, and I just don’t accept that it [minimum unit pricing] will make any great difference to people’s behaviour.

## Discussion

This study has investigated the views of policy stakeholders on the use of econometric modelling to inform a high-profile public health policy debate. We found individuals involved in policy debates around MUP in Scotland and/or the UK felt familiar with modelling studies and in many cases, displayed a detailed understanding of the Sheffield model in particular. Despite this, many were uneasy about the extent to which the Sheffield model could be relied on as knowledge for informing policymaking and largely preferred traditional evaluations. A tension was identified between this preference for evaluations and a desire for evidence based on the analysis of local, and therefore potentially more relevant, data sets. The modelling was therefore viewed as offering a higher external validity than the evaluations that are based on data from elsewhere. However, some expressed concern that the Sheffield model did not adequately capture the ‘real life’ world of the alcohol market, which was conceptualized as a complex and, to some extent, intrinsically unpredictable system—echoing issues debated within the academic literature^25^^,^^26^ Communication of modelling results to the varied audiences involved in the public policy debate was often viewed as suboptimal but also considered intrinsically difficult. Presenting an appropriate picture of the uncertainties inherent in modelling was viewed as necessary. There was enthusiasm for increased use of modelling to inform public health policy but an appreciation such evidence should only form one input into the process.

Our study has a number of strengths. We have carried out in-depth interviews with a broad range of policy stakeholders to elicit views based on real-life experiences rather than hypothetical scenarios. We have achieved good coverage for our groups of interest and key individuals. In addition, by interviewing those involved in the more advanced Scottish as well as UK policy debates, we have been able to consider the influence of the stage of the policy debate to some extent. However, a number of limitations must be noted. In such a highly politicized area, interviewee responses were inevitably influenced by political context and their viewpoint on MUP. To obtain a rich understanding, we have focussed on one specific policy debate and so views on econometric modelling may differ in other contexts. We have deliberately not sought to describe detailed criticisms specific to the Sheffield model because these are less likely to have transferable implications but their importance was considered within the analysis. However, we note the active debate in the academic literature^27–31^ and in industry-funded and other reports.^32–34^

While health service and health systems decision-makers’ views on modelling studies have been examined,^35–37^ there has been little research on the use of similar methods for informing public health policy outside the health sector. In keeping with our results, previous research on health technology assessments has found that those responsible for considering modelling evidence often display a good understanding of the principles underpinning the models but difficulties remained in communicating the level of uncertainty and the importance of assumptions underlying modelling evidence.^36^ Similarly, a qualitative study of the views of policymakers (within the UK’s National Health Service) on cardiovascular disease modelling echoed some of our findings.^37^ For example, the authors found both qualified support for increased use of modelling for a range of purposes (including predicting the impacts of population-based interventions) and concerns about the level of complexity incorporated into modelling studies.

Recent experience with pandemic influenza illustrates that modelling cannot be considered a panacea.^38^^,^^39^ In the early phases of the pandemic, mathematical models were used by policymakers to help predict the future burden of disease but have been subsequently found to have overestimated the real-life impact (although some difference would be expected as a result of changes in decision-making occurring in response to the model). However, econometric models have been helpful in other areas, such as tobacco control.^40^

The experience of the Sheffield model, with its use of local data to predict the effects of specific policy options, may serve as a template for public health professionals and researchers in other policy areas seeking to influence policy development through the application of public health evidence. Policy stakeholders appear willing to engage with such evidence, but there remains a need for high-quality evaluations too.

## Funding

This study received no specific funding. At the time of the research, S.V.K. and L.B. were funded by the Chief Scientist Office at the Scottish Health Directorates as part of the Evaluating Social Interventions programme at the MRC/CSO Social and Public Health Sciences Unit (25605200 68093). S.H. is funded by the Medical Research Council as part of the Understandings and Uses of Public Health Research programme (25605200 68096).

*Conflicts of interest*: All authors are investigators in an NIHR-funded evaluation of the impacts of the introduction of minimum unit pricing of alcohol in Scotland. They have no other conflicts of interest.

**Key points**

- There is often a lack of evaluation-based evidence on the effectiveness of many population-based public health policy interventions.
- Interest in the use of modelling studies to predict the effects of public health policies is growing, but little is known about policymakers’ views on their utility.
- Policy stakeholders involved in considering the introduction of a high-profile public health policy were comfortable in drawing on econometric modelling to inform decision-making, but many expressed a preference for traditional evaluation-based research.
- There is a willingness to make greater use of econometric modelling studies to inform public health policymaking, both for novel interventions and to provide contextually specific evidence, but these should complement, rather than replace, more traditional evaluation studies.
